# The Risk and Predictors of Malignancies in Ankylosing Spondylitis Patients in Israel—A Retrospective Electronic Data-Based Study

**DOI:** 10.3390/jcm12155153

**Published:** 2023-08-07

**Authors:** Polina Kagan, Noy Horesh, Howard Amital, Avishai M. Tsur, Abdulla Watad, Arnon D. Cohen, Niv Ben-Shabat

**Affiliations:** 1 Department of Medicine B, Zabludowicz Center for Autoimmune Diseases, Sheba Medical Center, Tel-Hashomer 5262100, Israel; drpolinakagan@gmail.com (P.K.); yam.noy@gmail.com (N.H.); howard.amital@sheba.health.gov.il (H.A.); watad.abdulla@gmail.com (A.W.); 2 Sackler Faculty of Medicine, Tel-Aviv University, Tel Aviv 6997801, Israel; 3 Israel Defense Forces, Medical Corps, Tel Hashomer, Ramat Gan 9112102, Israel; 4 Department of Military Medicine, Faculty of Medicine, Hebrew University of Jerusalem, Jerusalem 7610001, Israel; 5 Section of Musculoskeletal Disease, Leeds Institute of Molecular Medicine, University of Leeds, NIHR Leeds Musculoskeletal Biomedical Research Unit, Chapel Allerton Hospital, Leeds LS7 4SA, UK; 6 Chief Physician’s Office, Clalit Health Services, Tel-Aviv 6209813, Israel; arcohen@clalit.org.il; 7 Siaal Research Center for Family Medicine and Primary Care, Faculty of Health Sciences, Ben-Gurion University of the Negev, Beer-Sheva 8410501, Israel

**Keywords:** ankylosing spondylitis, spondyloarthropathy, cancer, malignancy, DMARDs, anti-TNF, NSAIDs

## Abstract

Background: Previous studies demonstrated unclear and vast variability in the association between Ankylosing Spondylitis (AS) and the risk of cancer. Objectives: To assess the risk of overall and site-specific malignancies for AS patients in Israel, while examining the role of comorbidities and immunomodulatory therapy. Methods: We conducted a retrospective electronic data-based study including all AS patients diagnosed between 2002 and 2018, with no history of cancer prior to enrollment, with 5:1 ratio matched-control by age, gender, and place of residence. The odds Ratios (OR) for site-specific malignancies, comparing AS patients and controls, were calculated using logistic regression. Risk factors for malignancies within the AS cohort were evaluated in the same manner. Results: This study comprised 5825 AS patients and 28,356 matched controls. There was a higher overall risk of cancer in AS patients compared to controls (OR = 1.4, 95% CI 1.24–1.6), specifically for solid malignancies (OR = 1.5, 95% CI 1.3–1.7), CNS (OR = 3.72, 95% CI 1.29–10.7), kidney (OR = 2.06, 95% CI 1.12–3.8), and malignancy of unknown primary (OR = 3.06, 95% CI 2.35–3.98). Regarding predictors for malignancy within AS patients, older age at diagnosis (OR = 1.31, 95%,CI 1.25–2.36), diabetes (OR = 1.52, 95% CI 1.18–1.97), IBD (OR = 2.61, 95% CI 1.75–3.89), and treatment with DMARDs (OR = 2.17, 95% CI 1.65–2.83) were associated with a higher risk of solid malignancies, while NSAIDs treatment alone had a protective effect for solid malignancies (OR = 0.78, 95% CI 0.61–0.99). No significant association was found between anti-TNF therapy and the risk of solid or hematologic malignancies within the AS group. Conclusion: AS is associated with an increased risk of overall and site-specific malignancies, with independently higher risk for older age, comorbidity of DM, IBD, and treatment with DMARDs.

## 1. Introduction

Ankylosing Spondylitis (AS) is a chronic, inflammatory, rheumatic disease primarily involving the spine and sacroiliac joints and is part of the spondylarthritis (SpA) group of diseases, with an approximate prevalence of 0.9% worldwide [1]. In addition to the articular manifestations, AS is associated with a higher incidence of anterior uveitis, inflammatory bowel disease (IBD), and psoriasis [2]. Cancer is one of the two leading causes of mortality and is a significant cause of morbidity in Israel and developed countries [3]. Despite the advances in primary prevention in recent decades, the overall cancer incidence is far from being controlled [4]. The association between chronic inflammation and tumorigenesis has been widely investigated [5]. In 1863, Virchow first suggested that cancer may initiate from the loci of chronic inflammation, known as the chronic irritation theory [6]. Not only organ-localized inflammation but also systemic inflammation seems to be linked to the risk of malignancy, as shown in some autoimmune diseases [7], with a higher incidence of both hematological and non-hematological malignancies in Sjogren’s syndrome (SJS), rheumatoid arthritis (RA), systemic lupus erythematosus (SLE), and giant cell arthritis (GCA) [8,9,10,11].

However, the association between AS and cancer is unclear. Different cohort studies in the past observed an inconclusive association between AS and the risk of malignancy. These heterogeneous results could be related to the variance of a geographic region with genetic heterogenicity, differences in the prevalence of cancer, study design, or sample size. Among the few studies that addressed the risk for specific types of cancers, higher rates of solid malignancies including colon, pancreatic, bone, and prostate cancer, as well as hematological malignancies such as lymphoma and multiple myeloma, were found to exist among AS patients [12,13,14].

In this study, we aimed to assess the risks of overall and site-specific malignancies in Israeli patients with AS compared to the general population and investigate risk factors for malignancy with AS patients—based on demographic, pre-existing comorbidities, and immunomodulatory therapy related to the disease.

## 2. Materials and Methods

### 2.1. Study Population and Design

This retrospective electronic data-based study compared AS patients to a cohort of matched controls without AS. All individuals newly diagnosed with AS (ICD-9 codes 720.0) with at least one documented diagnosis made in primary care centers, inpatients, and outpatient clinics, or hospitalization discharge letters between 1 January 2002 and 31 December 2018, were included. Five controls were randomly assigned for each case with matching for age, sex, and place of residence (within the district/neighborhood level). Controls were assigned an index date based on their AS match diagnosis date. Data were available up to 1 July 2019.

### 2.2. Data Source

The Clalit Healthcare Services (CHS) Ethics Committee approved this study in Beer-Sheva, Israel (Approval number 0212-17). The data were retrieved from the CHS electronic database, Israel’s most significant health maintenance organization, which serves over half of the Israeli population (approximately 4.5 million insured members) from heterogeneous ethnic groups and has continuous input from pharmaceutical, medical, and administrative operating systems. The database is used for administrative and clinical management and is available for epidemiological research. In addition, patient data can be automatically extracted from the database using data-mining techniques. The CHS database was previously used and validated in other studies made by our group on AS patients in Israel [15].

### 2.3. Variables and Measures

The definition of malignancy was based on a documented diagnosis in the medical records, as registered in the CHS database, based on ICD-9 codes. A subject was considered to have a malignancy if having a documented diagnosis of any cancer in the CHS database. The variable “overall cancer” is defined as having at least one malignant condition, either solid or hematological. For each subject, age, sex, ethnicity, body mass index (BMI), and socioeconomic status (SES) at the follow-up were obtained from their medical records in the CHS database. Age was considered as the age at the AS diagnosis, divided into four groups (18–34, 35–54, 55–75, >75). The SES was defined according to the poverty index of the member’s residence area, as defined in the 2008 National Census. Specifically, the poverty index was computed based on household income, education, marital conditions, and car ownership. Based on cluster analysis, it ranges from 1 to 20, with 1 being the lowest SES and 20 the highest. In our study, these layers were divided into terciles (low, medium, and high) [16]. BMI was dichotomized into over and under 30 kg/m^2^. As the likelihood of developing cancer may be confounded by other risk factors, traditional comorbidities such as smoking (ever/never), hypertension (HTN), and diabetes mellitus (DM) at baseline were also obtained and analyzed. Other comorbidities known to be more prevalent in AS patients, such as IBD and psoriasis, were also evaluated based on diagnostic codes from CHS datasets identified prior to the outcome of interest. Treatment was obtained from the medical records registered in the CHS database. It included nonsteroidal anti-inflammatory drugs (NSAIDs), disease-modifying anti-rheumatic drugs (DMARDs) such as Sulfasalazine and Methotrexate (MTX), and anti-tumor necrosis factor (TNF). We included the specific therapy with at least one documented dispense of the drug during follow-up.

### 2.4. Statistical Analysis

Differences in baseline characteristics between different groups of independent variables were compared using a t-test or Mann–Whitney U test for continuous variables and a χ^2^ test for categorical variables. Differences where *p* < 0.05 were considered statistically significant. The odds ratio (OR) for cancer was computed by comparing AS patients and controls, using logistic regressions, with a 95% confidence interval (CI). The Bonferroni test was used to correct alpha values to account for multiple comparisons. In addition, risk factors for malignancy within the AS cohort were also calculated similarly. The Hosmer–Lemeshow goodness-of-fit test was used to evaluate the models. The multivariate model accounted for age, gender, smoking, and distinct risk factors for cancer in specific malignancies. All data were analyzed using the Statistical Package for the Social Sciences; SPSS for Windows, V.26.0 (IBM SPSS Statistics).

## 3. Results

### 3.1. Demographic Characteristics

This study included 34,181 subjects: 5825 patients with AS and 28,356 controls. The baseline characteristics of the study population are presented in Table 1. The mean age was 50 years, with 63% of patients over the age of 50. AS patients had a significantly higher rate of DM (14.3% vs. 11.9%, *p* < 0.001), HTN (26.2% vs. 21.8%, *p* < 0.001), IBD (5.8% vs. 0.8%, *p* < 0.001), and psoriasis (5.1 % vs. 1.4%, *p* < 0.001), as expected.

### 3.2. Risk for Malignancy in AS Patients

The rates and risks for different malignancies, adjusted for age, sex, SES, and cancer risk factors, are presented in Table 2. In general, AS patients had a significantly increased risk of developing any malignancy compared to the control group (odds ratio (OR) 1.4, 95% CI 1.24–1.6), solid tumors (OR 1.5, 95% CI 1.3–1.7), CNS (OR 3.72, 95% CI 1.29–10.7), kidney (OR 2.06, 95% CI 1.12–3.8), and malignancy of unknown primary (OR 3.06, 95% CI 2.35–3.98).

For these selected malignancies with a greater prevalence in the AS population, the mean age (years ± SD) at the diagnosis of malignancy was compared between study groups and presented in Table 3. Patients with AS were diagnosed significantly earlier with solid tumors (70 ± 17.9 versus 74.4 ± 16.2, *p* < 0.01) and with malignancy of unknown primary (60.5 ± 17.2 versus 67.3 ± 17.4, *p* < 0.05) compared with the controls. In addition, the mean age for diagnosing overall cancer and kidney cancer was higher in AS patients compared to the control (82.5 ± 17.9 versus 74.8 ± 11.3, *p* < 0.05) (considering the Bonferroni correction for multiple comparison α = 0.002).

### 3.3. Independent Predictors for Malignancies in AS Patients

Within the AS group, an older age at diagnosis of AS (for every five years, OR 1.31, 95% CI 1.25–2.36), diabetes (OR 1.52, 95% CI 1.18–1.97), and IBD (OR 2.61, 95% CI 1.75–3.89) were significantly associated with higher rates of solid malignancies, while low SES was associated with lower rates of solid malignancies (OR 0.44, 95% CI 0.27–0.73). Regarding treatment, AS patients treated with only NSAIDs had a lower risk of developing solid malignancies (OR 0.78, 95% CI 0.61–0.99) opposed to those treated with DMARDs, such as MTX (OR 2.17, 95% CI 1.65–2.83) and Sulfasalazine (OR 1.58, 95% CI 1.2–2.09), which demonstrated a higher risk for these tumors. There was no significant association between anti-TNF therapy and the risk of solid malignancies (OR 1.2, 95% CI 0.91–1.6) or hematologic malignancies (OR 0.57, 95% CI 0.25–1.3). For hematological cancers, only older age at diagnosis of AS (OR 1.04, 95% CI 1.03–1.05) was a significant predictor (Table 4).

## 4. Discussion

In the present study, we have demonstrated a greater risk of cancer or solid malignancies among patients with AS. Kidney, CNS, and cancer of unknown primary were found to be more prevalent in patients with AS than in controls, with no significant effect on hematologic malignancies. Among AS patients, older age, diabetes, and IBD were associated with higher rates of solid malignancies, while low SES demonstrated an opposed trend. Treatment with DMARDs was associated with a higher risk for solid malignancies compared to NSAIDs treatment alone, which demonstrated a protective effect for solid malignancies. AS patients treated with anti-TNF therapy showed no excessive risk for solid or hematologic malignancy.

Nevertheless, the association between AS and malignancies is equivocal. Our study found that, overall, AS patients are 40% more likely to develop any type of cancer. To the best of our knowledge, this is the first large cohort study that shows the prevalence of the different types of malignancies in AS patients compared to the general population in Israel. The study by Sun et al. [17], conducted in 2014, included 4133 AS patients who reported the same trends in men and women separately. Later, other studies from Taiwan’s National Health Insurance database in 2017 and 2021 demonstrated the same association [12,18]. Similarly, the first meta-analysis by Deng and Li [14] included 23 studies that showed a 14% increase in the overall risk of malignancy (RR 1.14, 95% CI 1.03–1.25), with a significant association for Asian populations only in subgroup analysis. More recent studies also showed the same association as a cohort study conducted in 2019 of Korean males including 21,780 AS patients, with a 25% higher risk of cancer observed (SIR 1.25, 95% CI 1.15–1.36), with significant results found when divided into solid and hematologic malignancies [13]. In addition, a study by Walsh and colleagues in 2018 [19] that included patients from the United States also supported this conclusion. However, Swedish studies [20,21] found no increased risk of solid malignancies or lymphomas in patients with AS compared with the general population. This difference may be explained by the small number of cancer cases observed in the studies, affecting the significance of the results. Moreover, studies in Asia showed a positive relationship between AS and malignancy, while no association was found in studies of a Scandinavian population. Therefore, it should raise the question whether there is an association between geographic location and race/ethnicity with the risk of developing malignancy in the AS population. This variability in malignant risk underlines the importance of this study conducted for the first time in the Israeli population.

Within the AS cohort, pre-existing conditions of DM, HTN, IBD, and psoriasis are more prevalent than in the general population. Several studies have shown the association between these conditions and the presence of AS [22,23]. Patients with AS are more restricted in their motion and have a sedentary lifestyle because of their axial involvement and consequent pain. Hence, decreased exercise can contribute to developing DM and HTN [23,24]. In addition, there is a known association between AS, IBD, and psoriasis [23,24]; in our study, IBD and psoriasis were 7.5 and 3.5 times higher in AS patients than in the general population, respectively.

Our study demonstrated no excessive risk for hematological malignancies in the AS group (OR 0.75, 95% CI 0.52–1.1). When sorted by age, a slightly higher risk of hematological malignancy was found in elderly AS patients (OR 1.04, 95% CI 1.03–1.05). As known, many hematological malignancies may have a prolonged latent phase, with an increasing risk in older age [25], and these results may be related to non-prolonged-enough follow-up or non-old-enough cohort participants in our study, as more than 60% of participants were under 55 years old, with a mean follow-up of 10 years.

Given the risk of specific solid malignancy amongst AS patients, a large variety can be seen in the studies. Our study demonstrated a specific increased risk of cancer originating from the kidney, CNS, and of unknown primary. We observed a two-time greater risk of renal malignancy (OR 2.07, 95% CI 1.13–3.80). Similar results were found in a study from 2020 that showed a 25% higher risk of renal cell cancer (OR 1.25, 95% CI 1.23–1.28) [26]. One hypothesis pointed to the frequent exposure to X-ray examination of the pelvis in AS patients as the causative agent [27]. Another explanation is that AS, as an immune-mediated disease, results in a higher grade of inflammation, which is a risk factor for urological cancer [28]. IBD has a strong association with AS. A meta-analysis published in 2020, conducted by Feng and Bai [29], showed an additional increased risk of renal cancer, especially for patients with Crohn’s disease. A possible mechanism suggested in that study is the carcinogenic effect of longstanding use of immunomodulatory therapy (similar to the one given to AS patients) [30]. The confounding effect of IBD is likely to explain our results as this variable was adjusted.

To the best of our knowledge, the excessive risk of CNS malignancy and cancer of unknown primary with AS patients is reported here for the first time. In contrast, a differing conclusion was achieved in a study conducted in Sweden 2015 [31], which examined the risk of cancer of unknown primary for patients with autoimmune diseases. Their conclusion pointed to a decreased risk of cancer of unknown primary for male patients with AS only (SIR 0.49, 95% CI 0.23–0.9), while their overall finding was compatible with our results, showing an elevation of the overall risk among patients with autoimmune diseases (SIR 1.27, 95% CI 1.22–1.32). One theory suggested in their study was the possible protective effect of NSAIDs therapy against cancer, which was abundantly given to AS patients as a standard medication (shown in our research). NSAIDs were shown to have a protective effect on the emergence of solid malignancies (OR 0.78, 95% CI 0.61–0.99). This effect was previously reported in other epidemiological studies, by Kune et al. [32], who reported a reduction of 40% in the risk of colon cancer with the daily use of aspirin among users and non-users. Other studies have shown similar trends for non-gastrointestinal cancer and long-term use of aspirin [33,34]. Furthermore, the relationship between chronic inflammation and cancer has long been discovered [35]; therefore, it is logical to believe that drugs that inhibit inflammation, such as NSAIDs, may be beneficial in the prevention of cancer. Apart from their anti-inflammatory properties, possible mechanisms which may play a role in the anti-cancer effects of NSAIDs include their ability to induce apoptosis, inhibit angiogenesis, and enhance cellular immune responses [36].

Contrary to the trend we found for NSAIDs therapy alone, treatment with DMARDs (MTX and Sulfasalazine) was associated with a higher risk of solid malignancies (OR 2.17, 95% CI 1.65–2.84; OR 1.58, 95% CI 1.20–2.09, respectively). We found no other studies examining this association in AS patients. Psoriatic arthritis (PsA) is known to share biological, genetic, and clinical characteristics with AS [37]. In a systematic review and meta-analysis by Luo and Deng [38], there was an increased risk of cancer among patients with PsA treated with conventional synthetic DMARDs (pooled RR 1.75, 95% CI 1.40–2.18).

Although the risk of malignancy using anti-TNF agents was observed in RA in the past (especially for lymphomas) [39,40], our study did not find any significant effect for anti-TNF therapy on malignancies in the AS population with anti-TNF-naïve patients (OR 1.25, 95% CI 0.91–1.6). Similar conclusions were reached by other studies [40,41,42]; Bonovan’s study in 2016 [40], a systematic review and meta-analysis of 32 randomized control trials (RCTs), showed no significant evidence of an association between anti-TNF therapy and cancer risk in PsA and AS patients in 10 years follow-up. In addition, the same results were found in a study that observed the long-term safety of adalimumab treatment, conducted by Burmester, on 23,458 patients with rheumatic diseases, including AS Patients [42]. Nevertheless, these insignificant results may be related to a small number of anti-TNF therapy cases, which is represented in a wide confidence interval, or with a short follow-up period, and may not reflect the actual risk of malignancy in this group. Given the extensive use of TNF inhibitors for patients with AS, it is essential to continue monitoring the long-term risk profile for cancer in future studies.

Our study has several strengths. First, using a large cohort from a validated electronic database represents more than half of the Israeli population, allows better precision of estimates for different sub-groups, and increases the study’s external validity. Moreover, the database consisting of inpatients and outpatients with free medical insurance in Israel reduces the possibility of referral biases. Nonetheless, several significant limitations should be acknowledged. Our study’s AS diagnosis was based on ICD-9 codes, with no available data regarding clinical diagnostic criteria. The validity of AS diagnoses is strengthened by previous studies conducted using this database [15,43,44], and by the higher rates of IBD and psoriasis seen in the AS cohort. Additionally, although we required only one diagnostic documentation in the inclusion criteria for AS, de facto 95% of the study subjects had at least two diagnoses registered in their medical records. Another limitation is the lack of distinguishing and accurate drug indications for treatment in our database, as in the case of NSAIDs that can be sold as “over-the-counter” drugs. Therefore, some patients may use NSAIDs without registration in their medical files. Moreover, it is possible that patients treated only with NSAIDs have a less severe disease and have a lower risk of developing solid malignancies. Hence, it is not the treatment with NSAIDs that lowers the risk of solid malignancy but the lighter nature of the disease and patient’s characteristics.

In conclusion, our study demonstrated a greater risk of any cancer and solid malignancy among AS patients, specifically, kidney, CNS, and cancer of unknown primary. Within the AS group cohort, age, diabetes, and IBD were identified as independent risk factors for solid malignancies, while low SES demonstrated the opposite. AS patients treated with anti-TNF therapy did not demonstrate an excessive or reduced risk of solid or hematologic malignancies. Our findings emphasize the importance of maintaining the routine observation of patients with AS to identify the early development of cancer.

## Figures and Tables

**Table 1:** Baseline characteristics of the study population.

Characteristics	Controls (*n* = 28,356)	Ankylosing Spondylitis (*n* = 5825)
Age, years (mean ± SD)	48.93 ± 16.43	49.12 ± 16.56
18–35 (*n*; %)	6899 (24.3%)	1393 (23.9%)
36–55 (*n*; %)	11,226 (39.6%)	2305 (39.6%)
56–75 (*n*; %)	8253 (29.1%)	1693 (29.1%)
>75 (*n*; %)	1978 (7%)	434 (7.5%)
Gender (Female; %)	9815 (36.9)%	1965 (36.7%)
Ethnicity (non-Arab; %)	25,857 (97.3)%	5200 (97.3%)
SES (*n*; %) *		
Low	3931 (15.8%)	749 (15%)
Intermediate	17,340 (69.7%)	3493 (69.8%)
High	3614 (14.5%)	760 (15.2%)
Body Mass Index (*n*; %)		
<30 kg/m^2^	3009 (70.9%)	14,655 (76.3%)
≥30 kg/m^2^	1238 (29.1%)	4543 (23.7%)
Smoking (*n*; %)	8831 (33.2%)	1886 (35.3%)
Diabetes mellitus (*n*; %)	3171 (11.9%)	767 (14.3%)
Hypertension (*n*; %)	5788 (21.8%)	1403 (26.2%)
IBD (*n*; %)	207 (0.8%)	311 (5.8%)
Psoriasis (*n*; %)	381 (1.4%)	271 (5.1%)
Medications (*n*, %)		
NSAIDs	5648 (97%)	
NSAIDs only	3249 (55.8%)	
Anti-TNF	1760 (30.2%)	
DMARDs	1687 (29%)	
Sulfasalazine	1281 (22%)	
Methotrexate	956 (16.4%)	
Anti-TNF + DMARDs	1023 (17.6%)	

SD, standard-deviation; SES, socioeconomic status; IBD, inflammatory bowel disease; NSAIDs, non-steroidal anti-inflammatory drugs; TNF, tumor necrosis factor; DMARDs, disease-modifying anti-rheumatic drugs. * Available for 87.4% of data.

**Table 2:** Rates of malignancy during the study period, with a comparison between Ankylosing Spondylitis patients and controls.

Malignancy	Control (*n*, %)	AS (*n*, %)	Adjusted OR *	95% CI	*p*-Value
Any	1357 (5.1%)	375 (7%)	1.41	1.25–1.6	<0.001
Solid	1193 (4.5%)	348 (6.5%)	1.5	1.31–1.7	<0.001
Lung	51 (0.2%)	4 (0.1%)	0.38	0.13–1.05	0.63
Breast ^a^	245 (0.9%)	58 (1.1%)	1.17	0.87–1.58	0.288
CNS	8 (0.03%)	6 (0.1%)	3.72	1.29–10.72	0.015
Pharyngeal	21 (0.1%)	8 (0.1%)	1.85	0.82–4.19	0.138
Larynx	20 (0.1%)	5 (0.1%)	1.22	0.45–3.25	0.691
Thyroid	59 (0.2%)	16 (0.3%)	1.34	0.77–2.33	0.299
Esophagus	3 (0.01%)	1 (0.018%)	1.61	0.16–15.49	0.681
Colorectal	194 (0.7%)	39 (0.7%)	0.89	0.63–1.27	0.549
Stomach	30 (0.1%)	4 (0.1%)	0.64	0.22–1.81	0.402
Pancreas	7 (0.02%)	2 (0.03%)	1.41	0.29–7.78	0.669
Liver and bile duct	7 (0.02%)	0 (0%)	0	0	0.979
Kidney	35 (0.1%)	15 (0.3%)	2.06	1.12–3.8	0.019
Bladder	92 (0.3%)	27 (0.5%)	1.42	0.92–2.19	0.11
Prostate ^b^	194 (0.7%)	46 (0.9%)	1.12	0.8–1.57	0.513
Uterus ^a^	27 (0.1%)	5 (0.1%)	0.9	0.34–2.36	0.839
Cervical ^a^	8 (0.03%)	5 (0.1%)	3.16	1.03–9.7	0.44
Ovary ^a^	14 (0.1%)	2 (0.03%)	0.7	0.16–3.1	0.642
Bone	5 (0.018%)	0 (0%)	0	0	0.981
Sarcoma	36 (0.1%)	7 (0.1%)	0.94	0.41–2.12	0.888
Melanoma	93 (0.3%)	25 (0.5%)	1.34	0.86–2.1	0.19
Hematologic	191 (0.7%)	30 (0.6%)	0.76	0.52–1.13	0.182
ALL	12 (0.045%)	1 (0.018%)	0.4	0.05–3.10	0.384
CLL	32 (0.1%)	3 (0.1%)	0.45	0.13–1.47	0.186
Hodgkin’s lymphoma	34 (0.1%)	7 (0.1%)	1.02	0.45–2.31	0.953
Non-Hodgkin’s lymphoma	113 (0.4%)	21 (0.4%)	0.91	0.57–1.46	0.71
MDS	6 (0.02%)	2 (0.03%)	1.6	0.32–7.94	0.565
Multiple myeloma	21 (0.1%)	2 (0.03%)	0.46	0.10–1.96	0.294
Unknown primary	149 (0.6%)	91 (1.7%)	3.06	2.35–3.98	<0.001

CI, confidence interval; OR, odds ratio; AS, Ankylosing Spondylitis; CNS, central nervous system; ALL, acute lymphoblastic leukemia; CLL, chronic lymphoblastic leukemia; MDS, myelodysplastic syndrome. * The model included the following variables: age, gender, ethnicity, socioeconomic status, smoking, diabetes mellitus, inflammatory bowel disease, hypertension, and psoriasis; ^a^ Only females were included in the model; ^b^ Only males were included in the model.

**Table 3:** Mean age of selected cancer diagnosis in the study population.

	Age at Malignancy Diagnosis (Years)		
Malignancy	Control (Mean ± SD)	Ankylosing Spondylitis (Mean ± SD)	*p*-Value
Any	73.3 ± 16.5	70 ±17.6	0.01
Solid	74.4 ± 16.2	70.2 ± 17.9	<0.001
Central nervous system	55.6 ± 16.9	61.8 ± 18.4	0.523
Kidney	74.8 ± 11.3	82.5 ± 17.9	0.035
Unknown primary	67.3 ± 17.4	60.5 ± 17.2	0.003

SD, standard deviation.

**Table 4:** Independent predictors for solid and hematologic malignancies in Ankylosing Spondylitis patients.

Characteristics	Solid			Hematologic		
	OR	95% CI	*p*-Value	OR	95% CI	*p*-Value
Age at diagnosis (every 5 years increment)	1.31	1.27–1.36	<0.001	1.04	1.03–1.05	<0.001
Gender	1.23	0.98–1.55	0.07	0.78	0.6–1.01	0.064
BMI	0.99	0.97–1.01	0.608	0.98	0.96–1.01	0.315
Low SES	0.45	0.27–0.73	0.001	No data available		
Ethnicity	0.81	0.38–1.71	0.586	1.26	0.64–2.46	0.497
Smoking	0.89	0.69–1.14	0.369	0.91	0.70–1.2	0.527
Diabetes mellitus	1.52	1.18–1.97	0.001	1.05	0.77–1.43	0.747
Hypertension	0.95	0.73–1.23	0.728	1.27	0.95–1.7	0.097
IBD	2.61	1.76–3.89	<0.001	0.56	0.53–3.17	1.3
Psoriasis	1.38	0.85–2.23	0.188	1.39	0.68–2.83	0.357
NSAIDs	0.78	0.61–0.99	0.044	1.14	0.79–1.63	0.48
Anti-TNF	1.2	0.91–1.6	0.184	0.57	0.25–1.3	0.183
DMARDs						
MTX	2.16	1.65–2.83	<0.001	1.62	0.96–2.75	0.071
Sulfasalazine	1.58	1.20–2.09	0.001	0.63	0.28–1.43	0.275

CI, confidence interval; OR, odds ratio; BMI, body mass index; SES, socioeconomic status; IBD, inflammatory bowel disease; NSAIDs, non-steroidal anti-inflammatory drugs; Anti-TNF, anti-tumor necrotizing factor; DMARDs, disease-modifying anti-rheumatic drugs; MTX, Methotrexate.

## Data Availability

Data is not publicly available due to the CHS privacy policy.
